# Hospital service areas – a new tool for health care planning in Switzerland

**DOI:** 10.1186/1472-6963-5-33

**Published:** 2005-05-09

**Authors:** Gunnar Klauss, Lukas Staub, Marcel Widmer, André Busato

**Affiliations:** 1 Institute for Evaluative Research in Orthopaedic Surgery (IEFO), University of Bern, Stauffacherstrasse 78, CH-3014 Bern, Switzerland

## Abstract

**Background:**

The description of patient travel patterns and variations in health care utilization may guide a sound health care planning process. In order to accurately describe these differences across regions with homogeneous populations, small area analysis (SAA) has proved as a valuable tool to create appropriate area models. This paper presents the methodology to create and characterize population-based hospital service areas (HSAs) for Switzerland.

**Methods:**

We employed federal hospital discharge data to perform a patient origin study using small area analysis. Each of 605 residential regions was assigned to one of 215 hospital provider regions where the most frequent number of discharges took place. HSAs were characterized geographically, demographically, and through health utilization indices and rates that describe hospital use. We introduced novel planning variables extracted from the patient origin study and investigated relationships among health utilization indices and rates to understand patient travel patterns for hospital use. Results were visualized as maps in a geographic information system (GIS).

**Results:**

We obtained 100 HSAs using a patient origin matrix containing over four million discharges. HSAs had diverse demographic and geographic characteristics. Urban HSAs had above average population sizes, while mountainous HSAs were scarcely populated but larger in size. We found higher localization of care in urban HSAs and in mountainous HSAs. Half of the Swiss population lives in service areas where 65% of hospital care is provided by local hospitals.

**Conclusion:**

Health utilization indices and rates demonstrated patient travel patterns that merit more detailed analyses in light of political, infrastructural and developmental determinants. HSAs and health utilization indices provide valuable information for health care planning. They will be used to study variation phenomena in Swiss health care.

## Background

Switzerland hosts the second most expensive health care system worldwide with health care expenditures accounting for approximately 6.2% of the GDP in 2001 [1]. In the same year overall cost of in-patient care reached 13.9 billion € with an average of 5800 € per case [2]. In order to curb costs, novel approaches to health care planning need to be assessed. We therefore investigated utilization patterns of hospitals and created population-based hospital service areas (HSAs). Such service areas take into account patient travel patterns and demonstrate how hospitals are utilized. They are a suitable area model for the analysis of variations in hospital use and health care expenditures. The topic is of national importance and should be of interest to other European countries, because the underlying methodology has proved to be an influential tool in health service research, especially in the United States [3-7].

Hospital and health service areas have been widely used to demonstrate variations in health care utilization rates and health care expenditures [8-26]. The large variation often cannot be attributed to underlying differences in the prevalence or incidence of diseases. Practice style differences, diagnostic and therapeutic uncertainties in clinical medicine and supplier-induced demand are the most widely employed hypotheses to explain these variations [27]. Especially the supplier-induced demand hypothesis makes it apparent that resource allocations should not ignore variation patterns: Two hypothetical regions might both maximally utilize local resources for a given diagnostic procedure. This taken by itself could wrongfully indicate the same need for granting further resources when in fact one region represents a very high per-capita procedure rate and the other region a very low per-capita rate. The procedure rate in the first region might be driven by supplier-induced demand. If this has led to an oversupply the granting of further resources is questionable. The example demonstrates the importance to incorporate knowledge of variation profiles into resource allocation decisions.

Measuring variation profiles requires suitable area models as units of analysis. Although it is tempting to rely on administrative areas, factors that influence hospital use – such as natural geographic boundaries, travel time, infrastructure and attractiveness of health services – may be independent of administrative areas (e.g. borough, county, or canton)[6]. Boarder-crossing residents who seek health care outside their own residential area make interpretation of low or high per-capita rates problematic due to a numerator-denominator mismatch [28]. Therefore the concept of health service areas was introduced. Several methods to form such areas are described in the literature [6,22,29] but only Wennberg's approach specifically accounts for patient utilization patterns [10]. His method creates areas according to the use of health services by a given population and is considered most suitable to study variation phenomena [22,28].

This paper discusses the methodological underpinning for creating population-based hospital service areas (HSAs), describes the steps to create them and characterizes each HSA geographically, demographically and by various health utilization indices and rates.

## Methods

### Data

We utilized federal discharge data from the Swiss hospital discharge master file from 1998 through 2001. This file was initiated in 1997. We chose a time period of four years to achieve stable estimates of discharge patterns and to avoid possible fluctuation over one-year periods. At the time of analysis no data later than 2001 were available for research purposes. The file contained over 4 million hospital discharges of persons residing in Switzerland. The unit of observation were individual hospital discharges. We excluded discharges of patients not living in Switzerland at the time of treatment.

Patient residences and hospital locations were recorded as census region which are in essence aggregated zip code areas. Switzerland is divided into 605 census regions; their precise name in the federal discharge dataset is 'MedStat-region'. These regions constituted the geographic building blocks of subsequent HSAs. Their creation accounted for demographic, socio-economic and geographic criteria to ensure uniformity and comparability. Two-hundred-and-fifteen census regions contained at least one hospital; we termed them hospital regions. Unfortunately, the Swiss hospital discharge master file does not contain zip codes for patient residence but only the respective MedStat-region. This limitation is attributable to strict confidentiality laws and is unlikely to change in the near future.

We obtained commercial GIS-compatible vector files for Swiss census regions [MicroGIS Ltd., Baar, Switzerland]. Age-, and sex-stratified population counts for each census region were derived from the 1990 census. Necessary age-, and sex-stratified population counts for each census region were not yet available from the 2000 census. The difficulty in obtaining these counts owes to the fact that census data are collected on a different area scale than the specific census regions used in the federal hospital discharge data file. Subsequent approximations, performed by the Swiss Statistical Office, require complex algorithms and make use of so-called hectare data sets. At the time of our analysis, these hectare data were not available from all cantons.

### Definition of health service areas

We defined hospital service areas through a patient origin study by cross-tabulating the sum of discharges of every residential region with all possible hospital regions. Each matrix cell gave the sum of discharges of residents from a given residential region in a hospital region. This step identified the main hospital provider region – the one with the highest number of discharges – for each residential region. A three-step algorithm first assigned residential regions to their main provider hospital region. We then adjusted several assignments manually to achieve contiguous hospital service areas. Contiguity, a geographical convention, ensures the readability and interpretability of maps [30]. Preliminary HSAs were finally checked for the plurality rule: whether the sum of all discharges of HSA-residents within their HSA was higher than the sum of discharges to any other HSA. Failed plurality necessitated merging HSAs. Assigning a unique colour to every residential region with the same hospital region graphically displayed newly defined HSAs.

### HSA characteristics

We characterized HSAs demographically, geographically, by health utilization indices, and rates. Our patient origin study provided four core variables for each HSA: (1) the population count; (2) the sum of discharges of HSA-residents irrespective of hospital location, i.e. including discharges in hospitals outside their residential HSA; (3) the sum of discharges of HSA-residents only in hospitals within their HSA; and (4) the sum of discharges in HSA-hospitals irrespective of residential HSA of patients, i.e., including patients living outside the HSA. These variables allowed the calculation of a series of discharge counts, health utilization indices, and health utilization rates. Depending on the denominator, health utilization indices can be sub-classified into population-based and hospital-based.

The localization index (LI) is the fraction of all discharges of HSA-residents that happened within their HSA. It is calculated by dividing discharges of HSA-residents within their HSA by the total discharges of HSA-residents. It is a population-based index and it indicates the degree of localization of hospital care provided for the population in a given HSA.

The inflow index (II) is the fraction of non-HSA-resident discharges of all discharges within HSA-hospitals. It is calculated by subtracting from 1 the quotient of non-HSA-residential discharges over the total number of discharges in HSA-hospitals. It is a hospital-based index that can be viewed as a crude measure of attractiveness. It also depends on the type of hospital services provided within a given HSA.

The market share index (MSI) is the fraction of HSA-resident discharges of all discharges within HSA-hospitals. It is calculated by dividing the number of residential HSA discharges by the total number of discharges in HSA hospitals. This too is a hospital-based index; it indicates the degree of localization of hospital care provision from a hospital perspective. MSI and II are complementary to each other; thus adding to one in each HSA. One is therefore sufficient and we favoured the II. Above indices can be multiplied by 100 to give percentages; we will present all indices as percentages.

The net patient flow (NPF) is the fraction of the overall resulting patient movement. It is calculated by dividing the sum of discharges in HSA-hospitals by the sum of discharges of HSA-residents, minus 1. A negative NPF indicates that more HSA-residents leave the HSA than non-resident patients move into the HSA for hospital services. A positive NPF indicates that the number of patients coming from outside for hospital services is greater than the number of HSA-residents leaving their HSA to receive hospital care. This index has no unit. To our knowledge the NPF is a novel measure which we calculated from a patient origin study.

We also used the four core variables to calculate the number of discharges of HSA residents that received hospital care outside their HSA, the number of discharges of non-HSA residents that received hospital care within a given HSA and the effective number of discharges that crossed HSA borders by subtracting the two discharge counts. Dividing the three counts by the population in each HSA and multiplying by 1000 gave three population based rates: the local-out rate quantifies the number of discharges per 1000 HSA-residents that receive hospital care outside their HSA, the nonlocal-in rate quantifies the number of nonresidents that are treated per 1000 HSA residents, and the net-rate quantifies the effective exchange of patients (either in or out) per 1000 HSA residents. Dividing each rate by the country-wide average provided a rate ratio to compare HSAs in terms of their patient movements.

### Data management/statistics

We performed data handling and statistical analyses in Stata 8^® ^[StataCorp., Texas, USA]; we used a GIS [ArcView 8.2^®^, ESRI, Redlands CA, USA] and vector files to map spatial data. This paper presents primarily descriptive statistics of characteristics of newly defined HSAs. We investigated the relationships among health utilization indices and among health utilization rates. Correlations of continuous variables with skewed distribution were assessed with Spearman's rho; two means were compared with the Student-t-test for normally distributed variables and the Wilcoxon rank sum test for skewed variables. We employed simple linear regression to analyse the extent of linear relationships between continuous variables.

## Results

### Swiss hospital service areas

**Figure 1:**
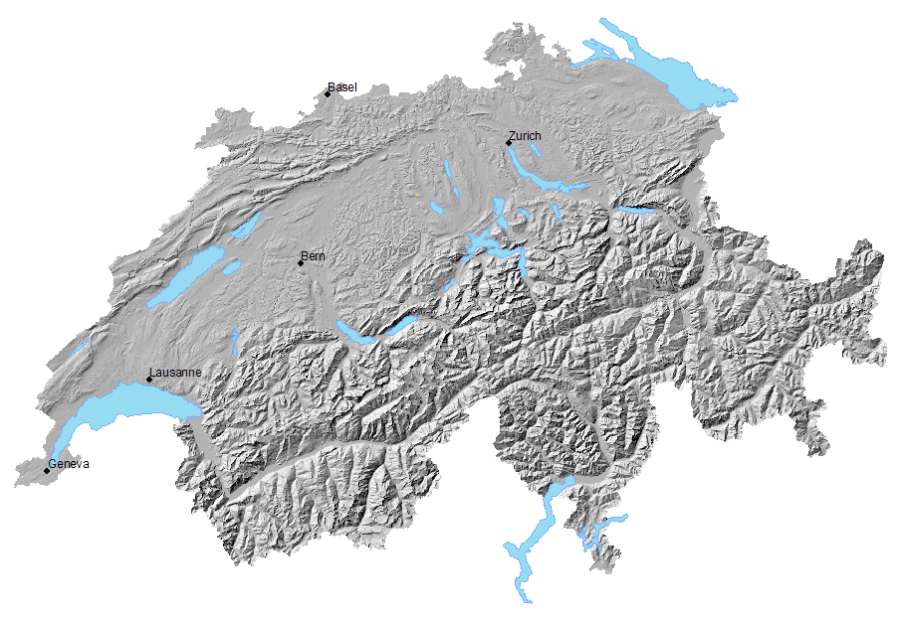
Swiss Topography.

**Figure 2:**
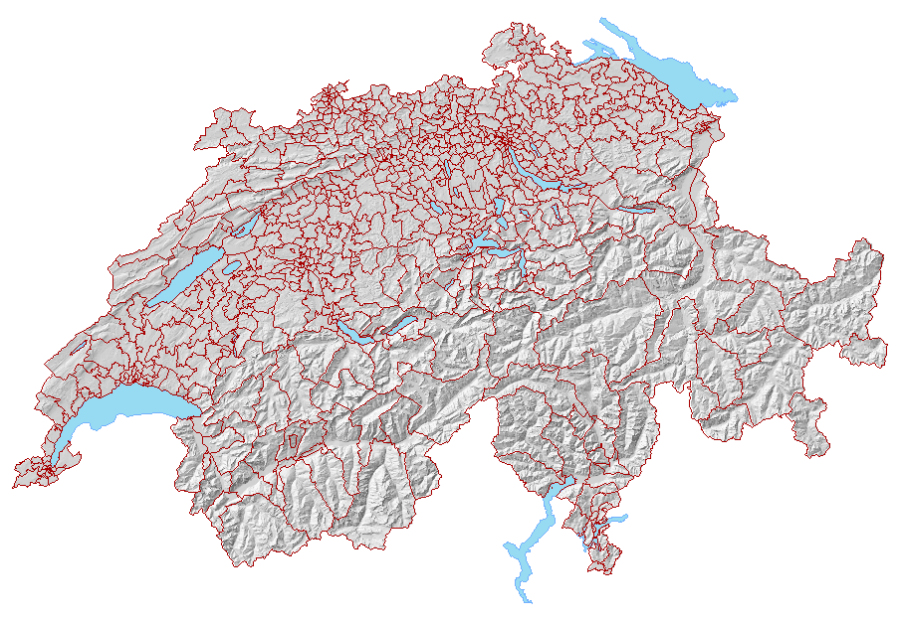
Census Regions; building blocks of Swiss HSAs.

Figure 1 gives the reader an overview of Swiss topography. The massive Alps run along the entire south and cover roughly half of the country's area. The Jura mountain range runs along the northwest. In between stretch the Swiss midlands, from southwest to northeast. They are densely populated and contain most urban centres. Figure 2 shows the 605 census regions, which are the building blocks for our HSA. We wish to point out the large census regions in the Alps and the Jura range. In comparison, census regions are smaller and more numerous in the Swiss midlands. Urban centres like Basel, Bern, Geneva, Lausanne, and Zurich are clearly recognizable.

**Figure 3:**
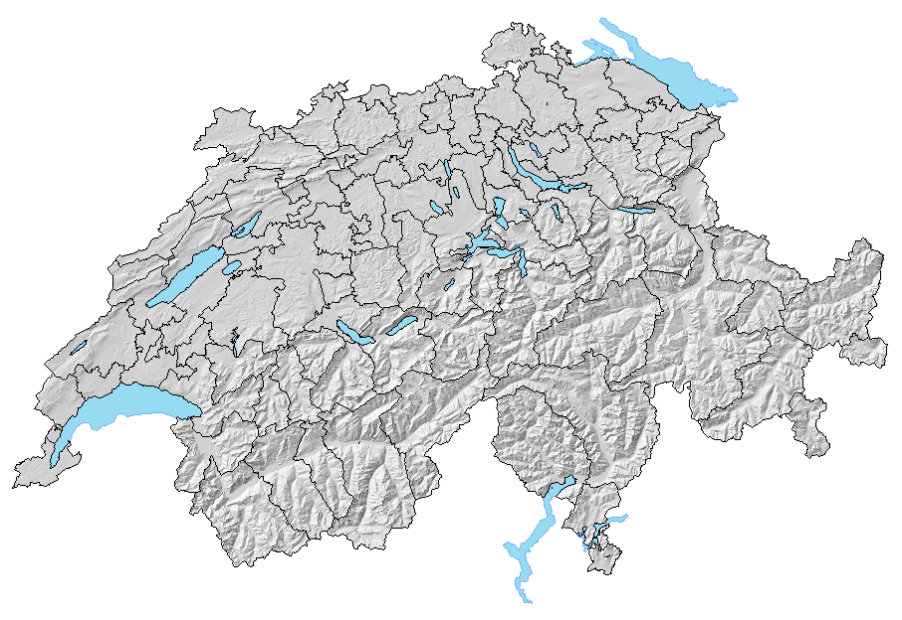
Swiss Hospital Service Areas.

The patient origin study yielded 100 HSAs as shown in Figure 3. Twenty two residential regions were reassigned for contiguity; one preliminary HSA was merged to achieve plurality. Fifteen HSAs incorporated a census region of a neighbouring canton which we defined as geographic extension over canton borders.

Table 1 gives an overview of core variables we retrieved from the patient origin study. HSAs were ranked according to the number of discharges of HSA-residents (population discharges). We then chose five HSAs with the highest ranks, five with the lowest ranks, and five with ranks around the median for demonstration. Table 2 gives; also for these 15 HSAs; the calculated discharge counts, health utilization indices, and health utilization rates to enables the reader replication of calculations.

**Table 1:** Core variables of patient origin study per HAS

**Lft**		**HSA**	**Pop**	**Pop_d**	**Local_d**	**Hosp_d**
1	GE20	Genève	375900	***70836***	67887	72637
2	VD13	Lausanne	266627	***53320***	42360	62779
3	BE09	Bern	365502	***46928***	41383	69197
4	ZH85	Zürich-Grünau	210799	***32178***	16803	37081
5	LU01	Luzern	224858	***30984***	25025	34019

51	SG11	Uznach	52679	***7418***	2659	2942
52	ZH12	Horgen	49791	***7383***	3729	5843
53	VS31	Sierre	38293	***6801***	4061	7074
54	SZ10	Schwyz	49187	***6415***	3512	4174
55	ZH04	Affoltern	35403	***6174***	3231	3635

96	GR03	Engiadina	6744	***1101***	683	852
97	BE67	Simmental	8806	***1037***	367	656
98	BE68	Oberhasli	8053	***1021***	642	2474
99	GR10	Poschiavo	4398	***727***	403	417
100	GR09	Val Müstair	1623	***250***	163	339

Legend of variable* abbreviations in Table 1

Pop Population

Pop_d Population discharges

Local_d Local discharges

Hosp_d Hospital discharges

* each variable is described in detail in the Methods section of the text

**Table 2:** Discharge counts, health utilization indices and health utilization rates retrieved per HSA

		**Discharge Counts**			**Indices**			**Rates**				
**Lft**	**HSA**	**L_out**	**NL_in**	**Diff**	**LI**	**II**	**NPF**	**Pop**	**Local**	**L_out**	**NL_in**	**Net**

1	GE20	2949	4750	1801	0.96	0.93	0.03	188	181	8	13	5
2	VD13	10960	20419	9459	0.79	0.67	0.18	200	159	41	77	35
3	BE09	5545	27814	22269	0.88	0.6	0.47	128	113	15	76	61
4	ZH85	15375	20278	4903	0.52	0.45	0.15	153	80	73	96	23
5	LU01	5959	8994	3035	0.81	0.74	0.1	138	111	27	40	13

51	SG11	4759	283	-4476	0.36	0.9	-0.6	141	50	90	5	-85
52	ZH12	3654	2114	-1540	0.51	0.64	-0.2	148	75	73	42	-31
53	VS31	2740	3013	273	0.6	0.57	0.04	178	106	72	79	7
54	SZ10	2903	662	-2241	0.55	0.84	-0.4	130	71	59	13	-46
55	ZH04	2943	404	-2539	0.52	0.89	-0.4	174	91	83	11	-72

96	GR03	418	169	-249	0.62	0.8	-0.2	163	101	62	25	-37
97	BE67	670	289	-381	0.35	0.56	-0.4	118	42	76	33	-43
98	BE68	379	1832	1453	0.63	0.26	1.42	127	80	47	227	180
99	GR10	324	14	-310	0.55	0.97	-0.4	165	92	74	3	-70
100	GR09	87	176	89	0.65	0.48	0.36	154	100	54	108	55

Legend of variable* abbreviations in Table 2

L_out Local-out discharges

NL_in Nonlocal-in discharges

LI Localization index

II Inflow index

NPF Net patient flow

Net Net rate

* each variable is described in detail in the Methods section of the text

### Demographic/geographic characteristics

HSAs showed marked differences in demographic and geographic characteristics. The mean population size was 68,867 (median 47,273), ranging from 1,623 to 375,900. Area size was on average 399 km^2 ^(median 311 km^2^), ranging from 35 to 2125 km^2^. Larger HSAs were more often seen in mountainous, decentralized regions. Thirteen HSAs consisted of a single census region; forty-one consisted of two, three, or four census regions. Combined they constituted 54% of HSAs. The number of hospitals per HSA ranged from one to sixteen, with 32 HSAs having only one serving hospital and 27 HSAs having five hospitals or more. HSAs with more than five hospitals were urban or contained agglomerations around cities. The ten HSAs with the highest population had on average larger area sizes and incorporated urban centres. The ten highest population densities were also seen in HSAs of large urban centres. The ten lowest population counts were seen in mountainous HSAs (irrespective of area size) and in smaller HSAs.

### Health utilization indices

**Figure 4:**
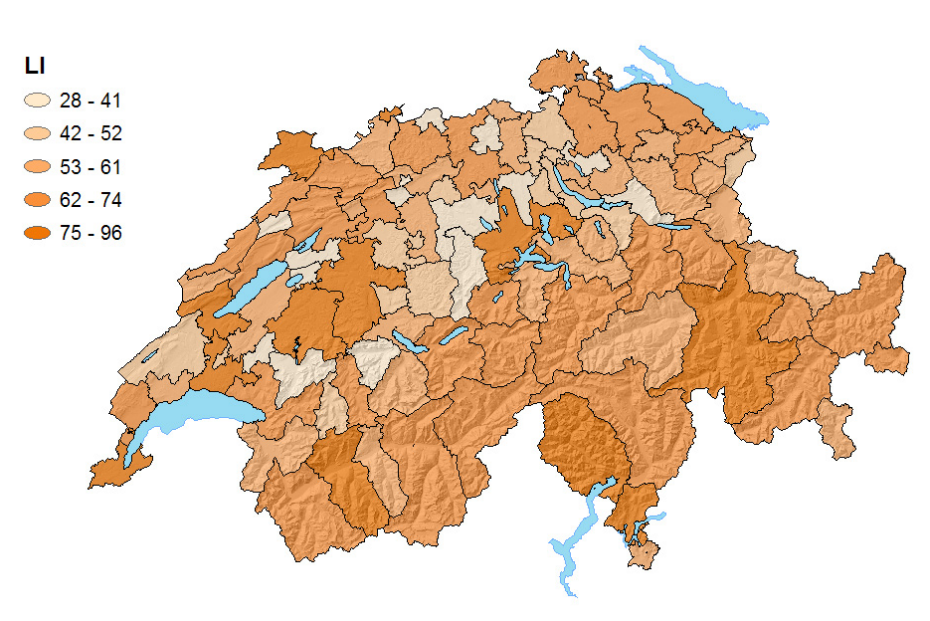
Localization Indices (in %) of Swiss HSAs.

Figure 4 depicts a map of the localization indices of HSAs. The HSA with the highest LI was Geneva (LI = 96%), the HSA with the lowest LI (of 28%) was situated in the Swiss Midlands. Three geographic patterns emerged: (1) HSAs of mountainous regions had above-average LIs; (2) HSAs with below-average LIs were predominantly highly developed region; (3) HSAs which incorporated any of the 14 large urban centres or agglomerations showed above-average LIs (mean = 75.7%) compared to the remaining HSAs (mean = 56.1%; p < 0.0001).

**Figure 5:**
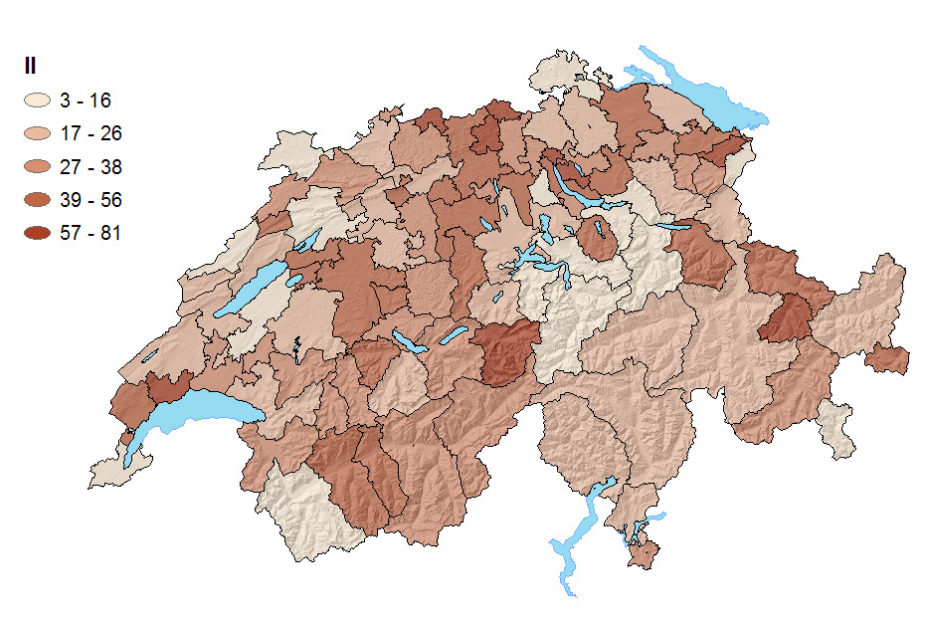
Inflow Indices (in %) of Swiss HSAs.

Figure 5 shows the inflow indices of HSAs as percentages. These ranged from 3% (mountainous HSA) to 81% (Swiss Midlands). A distinct geographic pattern is less easily discernible because high or low IIs could be found irrespective of demographic and geographic characteristics. We detected one weak geographic pattern: higher IIs were seen in a number of HSAs of urban centres, a distinct exception being the HSA of Geneva, which exhibits a very low II, combined with the highest LI. Other variables not discussed or measured in this study (e.g. hospital contracts, hospital specialties, rehabilitation centres) were likely more influential on the II of a given HSA.

**Figure 6:**
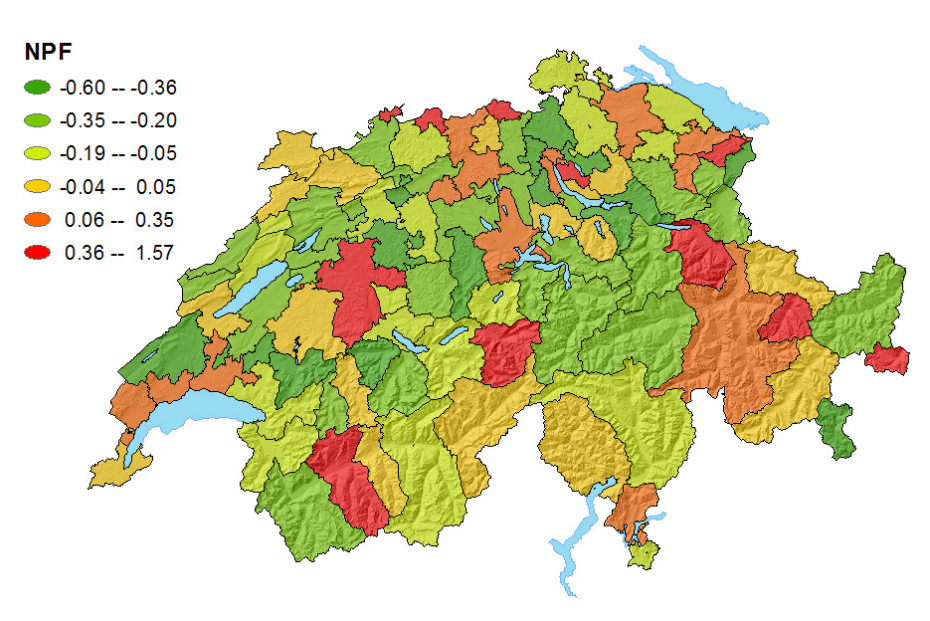
Patient Net Flow (Ratio) of Swiss HSAs.

Figure 6 shows the net patient flow of HSAs. Red colour indicates higher inflow of nonresident patients (NFP positive) than outflow of residents (NPF negative) to hospitals outside the HSA. Green colour indicates a higher overall outflow of residents. Yellow HSAs had a balanced NPF around zero (+/- 0.05). Most urban HSAs displayed high positive NPFs. HSAs surrounding the urban centres predominantly showed varying degrees of negative NPFs. Four mountainous HSAs with famous rehabilitation clinics (Davos, Meiringen, Crans-Montana, Walensee) also displayed high positive NPFs. Please be reminded that inflow of foreign patients is not considered in our study.

### Relationship of HSA utilization indices and utilization rates

**Figure 7:**
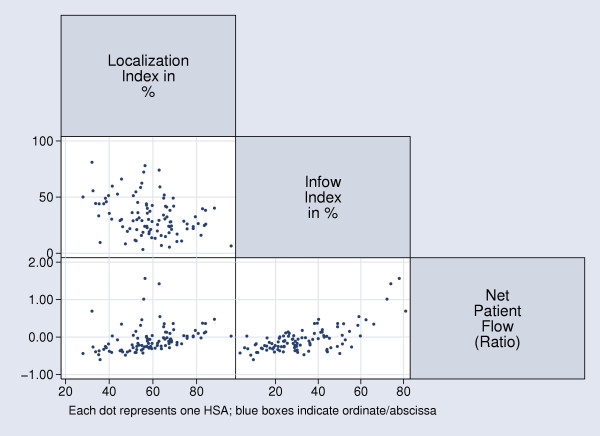
Correlation of Health Utilization Indices for Swiss HSAs.

We investigated the relationship among health utilization indices (Figure 7): LI, a population-based index, and II, a hospital-based index, demonstrated weak negative correlation (Spearman's rho = - 0.31; p = 0.018) which we decided to ignore (the corresponding graph appears rather like a cloud). Interestingly, NPF positively correlated with LI (Spearman's rho = 0.541; p < 0.0001), indicating that HSAs with higher degrees of localized hospital care also had a positive NPF or NPF around zero. HSAs with low localization of care, in comparison, had negative NPF with the exception of one outlier. II was, expectedly, positively correlated with NPF (Spearman's rho = 0.545; p < 0.0001).

**Figure 8:**
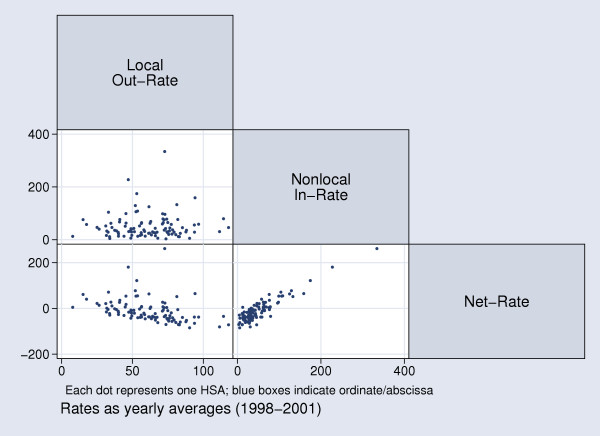
Correlation of Health Utilization Rates (per 1000 Residents) for Swiss HSAs.

We also investigated the relationship among health utilization rates (Figure 8): nonlocal in-rate and local out-rate were, not surprisingly, uncorrelated. A positive correlation of nonlocal in-rate and net rate (Spearman's rho = 0.76; p < 0.0001) as well as a negative correlation of local out-rate and net rate (Spearman's rho = -0.56; p < 0.0001) were also expected findings. Because both graphs indicated acceptable linear association, we regressed local out-rate on net rate (regression coefficient = -0.89, t = -3.94; p < 0.0001; R^2 ^= 0.13) and nonlocal in-rate on net rate (regression coefficient = 0.98, t = 21.65; p < 0.0001; R^2 ^= 0.82). Interestingly, net rate is driven slightly stronger by the inflow of nonresidents into an HSA than by the outflow of HSA residents to hospitals outside as indicated by the absolute values of the regression coefficients. Also, net rate is explained more consistently by the inflow of nonresidents into an HSA than by the outflow of HSA residents to hospitals outside as indicated by R^2^.

### Localization of hospital care

**Figure 9:**
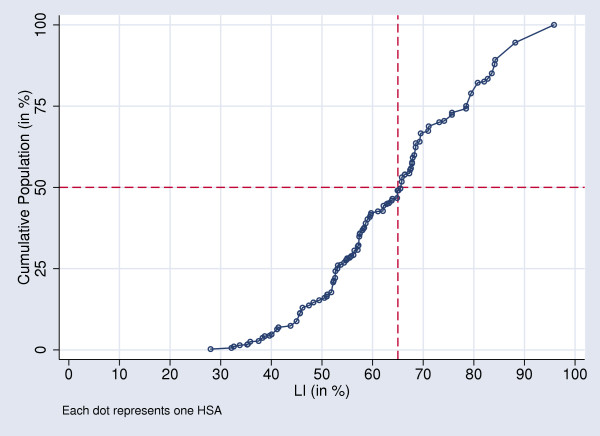
Cumulative Percentages of Swiss Population according to ascending HSA LI-ranks

The LI, a population-based index, gauges the tendency of patients within an HSA to use local hospitals. Plotting the cumulative population counts of HSAs against ranked LIs of HSAs visually demonstrated that 50% of the Swiss population lives in HSAs where at least 65% of hospitalizations occur locally (Figure 9). Likewise, about one fifth of the Swiss population lives in HSAs where less than 50% of hospitalizations occur locally and 25% live in HSAs with a localization of hospital care exceeding 80%.

## Discussion

### Data

In 1996 federal health statistical reporting was introduced in Switzerland. Public hospitals have a duty to disclose of medical, administrative, and economic data to the Swiss Federal Statistical Office. Five years after introduction hospital participation reached 99% with approximately 85% of public hospital admissions being documented [2]. All submitted individual-level data (e.g. hospital discharges) are subjected to an algorithm to check internal validity. Reliability of data is also enhanced by standardized documentation and data collection. The high data quality and completeness of documentation ensure reliability of our results.

### Methodology

We employed an established method to define hospital service areas derived from hospital discharge data [10,31,32]. An algorithm assigns residential areas to a hospital provider area in order to create populations congruent with respect to place of residence and use of hospital services.

Ideally, all hospital care for residents is provided within those residents' service area. As there are always patients seeking health care outside their region, the LI is a useful indicator of the validity of HSA definitions: High LIs are desirable and indicate congruent populations with respect to place of residence and hospital use. Likewise, patient net flow (PNF) and inflow from outside (II) should remain low. We observed moderate to high LIs and moderate to low IIs for the majority of HSAs. Nevertheless, 22 HSAs offered localized hospital care for less than 50% of residents and 15 HSAs had an inflow of patients living outside above 50%. Numerator-denominator mismatch might become a problem. It remains unclear whether the finding dispels this methodology because no hard criteria exist upon which to judge suitability. Health utilization indices may be used to judge reliability of utilization measures in the context of possible rate distortion, and to describe and understand patterns of health care use within and between HSAs.

HSAs must be sufficiently small to detect small area variation patterns. Yet, they need to be large enough to contain population sizes that will give utilization rates with acceptable reliability (i.e. sufficient number of observations). Balancing those competing interests is a challenge because area size is an important determinant of LI. Incorporating HSAs with low LIs into neighbouring HSAs to obtain a larger LI of the combined regions may fail to detect heterogeneous utilization patterns. Differences between two areas can be hidden in the overall rate. However, HSAs with low LIs may decrease the validity of per capita rates and make interpretation problematic. It needs to be shown whether a median population count of 47'273 and a median area size of 311 km^2 ^yield reliable per-capita rates for both common and less common medical conditions and surgical procedures.

We currently have no comparable data on health utilization indices for service areas obtained through different methods or for administrative areas. The LIs of 3436 hospital service areas used in the Dartmouth Atlas Project have a slightly larger range (17.9% to 94%) and the lowest LI was 10% lower than our lowest. It should be noted, however, that utilization rates in the Dartmouth Atlas are calculated for hospital referral regions (HRR) which are aggregated HSAs. HRRs are considerably larger than HSAs, thus increasing LI. The concept of HRR is not feasible for a small country like Switzerland.

Apart from the underlying area model, sizes and shapes of service areas are determined by the availability of hospital services and the actual utilization patterns of patients. Assuming that the area model and availability of hospital services were fixed variables for our study period, HSA size, shape and distribution were ultimately a function of hospital use by the population over four years. HSAs are thus not stable geographic constructs in time as hospitals close, merge, or open. Infrastructural changes, political regulations, and altered insurance policies may also necessitate patient origin studies with more recent discharge data. The speed of these changes will indicate appropriate intervals after which HSA definitions need to be updated.

### Literature

Various studies have defined health service areas using different methodological approaches [29,33,34]. Such service areas can be on a country [35], state, province [15,33] or even city [36-38] level, depending on the research question. In Europe a large body of literature has evolved around small area analysis [11,18,36-46], but to our knowledge no definition of population-based health service areas on a country level has been pursued to date. In The Netherlands the National Institute of Public Health and Environment has maintained an extensive web-based small area analysis project since 1999 [35]. Their area definitions are based on various spatial models, depending on subject matter. These areas are partly historical, not always geographically congruent, and to our knowledge not population-based.

### Limitations

The study has several limitations. These can be attributed to (1) the underlying area model, (2) the intrinsic diversity of hospital utilization, and (3) the combination of discharges irrespective of medical specialty.

Census regions, our underlying area model, were specifically created for the federal health statistics by aggregating zip codes areas. Being a less aggregated area model, zip codes might have yielded more precise estimates of the influence of patient utilization upon the size and shape of HSAs. Nevertheless, their use was precluded due to data confidentiality laws. The formation of census regions aimed to achieve comparability of socio-demographic and geographic factors. Despite their aggregated nature we consider census regions a reasonably valid area model to create HSAs.

We created HSAs using hospital discharge data irrespective of diagnosis or medical specialty. We wanted to give an overall view of hospital utilization. Determining HSAs by the totality of discharges may not reflect the utilization pattern of a population with a specific diagnosis or a specific age group [7]. There is a solution to this problem: calculated LIs of discharges with a specific diagnosis can be compared to LIs that were calculated from the overall discharge data. If the two LIs are similar for a given HSA, travel patterns can be assumed to be alike and the service area model is valid. If they are meaningfully different, service areas for the specific diagnoses or patient groups may have to be created. This will be necessary for highly specialized services like cardiac, spinal, and neurosurgery. Guagliardo et al. evaluated the appropriateness of the US Medicare-based HSAs from the Dartmouth project for paediatric discharges in California using this approach. They spoke of the fit of discharge data to the area model and calculated an LI "index divergence" [7]. It still needs to be established whether there is a scientific or health planning need for such sub-speciality HSAs in Switzerland.

### Implications

In the last decade the political structure of the Swiss health care system has come under scrutiny. The existence of 26 micro health systems, one for each canton, makes planning very complex. Patient movements over canton borders were estimated to exceed 13% in 2001. Nevertheless, such movements are not accounted for in the majority of planning processes. A canton-independent perspective for planning is therefore on the political agenda. In early 2004, the Cantonal Health Planning Board launched a study group of health care representatives from each canton plus federal representatives to assess novel approaches to hospital planning in Switzerland ["Arbeitsgruppe Leistungsorientierte Spitalplanung" der Gesundheitsdirektorenkonferenz]. Our institute collaborates with the above-mentioned board. HSAs are currently being reviewed for inclusion in a pending guideline on hospital planning strategies.

## Conclusion

Switzerland possesses ideal data to perform patient origin studies and define population based hospital service areas. The federal health statistics was formed specifically to monitor health care performance and provide data for epidemiologic studies. Our newly defined HSAs should function as units of analysis to assess regional distribution of health care resources and measure variation in utilization rates. They offer a finer discrimination than the traditional area model – canton – thus giving planners better inside into patient movements.

We are convinced that HSAs and the variables we derived from the patient origin study will lead to a better understanding of hospital use. Health utilization indices and rates provide new information on travel patterns and hospital use. They may be used to assess the current situation for a given region and for projections of future need of resources. Thereby HSAs will help to establish more awareness of differences in the use of hospital services. National benchmarks based on variation studies may unveil possible under- or over-use of resources.

## Abbreviations

GDP Gross domestic product

GIS Geographic information system

HRR Hospital referral region

HSA Hospital service area

II Inflow index

LI Localization index

MSI Market share index

NPF Net patient flow

SAA Small area analysis

## Competing interests

All authors declare that they have no competing interests. The study was partially funded by the Swiss national science foundation (SNSF grant 405340-104607/1).

## Authors' contributions

GK is responsible for drafting the manuscript. He obtained the hospital discharge data, carried out the statistics, and performed GIS operations. LS and MW substantially contributed to developing the final version of the manuscript. AB participated in the coordination of the study. All authors read and approved the final manuscript.

## Pre-publication history

The pre-publication history for this paper can be accessed here:



## References

[B1] OECD Health 2004, 3rd edition.

[B2] Swiss-Federal-Statistical-Office (2001). StatSanté - Informationen über das Projekt "Statistik der stationären Betriebe des Gesundheitswesens" [German/French].

[B3] Wennberg JE (1984). Dealing with medical practice variations: a proposal for action. Health Affairs.

[B4] Wennberg JE (1996). On the appropriateness of small-area analysis for cost containment.[comment]. Health Affairs.

[B5] Wennberg JE (2002). Unwarranted variations in healthcare delivery: implications for academic medical centres. Bmj.

[B6] Goody B (1993). Defining rural hospital markets. Health Serv Res.

[B7] Guagliardo MF, Jablonski KA, Joseph JG, Goodman DC (2004). Do pediatric hospitalizations have a unique geography?. BMC Health Serv Res.

[B8] Glover AJ (1938). The incidence of Tonsillectomy in School Children.. Proceedings of the Royal Society of Medicine.

[B9] Lewis CE (1969). Variations in the incidence of surgery.. New England Journal of Medicine.

[B10] Wennberg J, Gittelsohn (1973). Small area variations in health care delivery. Science.

[B11] McPherson K, Wennberg JE, Hovind OB, Clifford P (1982). Small-area variations in the use of common surgical procedures: an international comparison of New England, England, and Norway. New England Journal of Medicine.

[B12] Chassin MR, Brook RH, Park RE, Keesey J, Fink A, Kosecoff J, Kahn K, Merrick N, Solomon DH (1986). Variations in the use of medical and surgical services by the Medicare population. N Engl J Med.

[B13] Keller RB, Soule DN, Wennberg JE, Hanley DF (1990). Dealing with geographic variations in the use of hospitals. The experience of the Maine Medical Assessment Foundation Orthopaedic Study Group. Journal of Bone & Joint Surgery - American Volume.

[B14] Welch WP, Miller ME, Welch HG, Fisher ES, Wennberg JE (1993). Geographic variation in expenditures for physicians' services in the United States.[comment]. New England Journal of Medicine.

[B15] Gittelsohn A, Powe NR (1995). Small area variations in health care delivery in Maryland. Health Services Research.

[B16] Coyte PC, Hawker G, Wright JG (1996). Variations in knee replacement utilization rates and the supply of health professionals in Ontario, Canada. J Rheumatol.

[B17] Birkmeyer JD, Sharp SM, Finlayson SR, Fisher ES, Wennberg JE (1998). Variation profiles of common surgical procedures. Surgery.

[B18] Swart E, Wolff C, Deh S (2000). Häufigkeit und kleinräumige Variabilität von Operationen. Chirurg.

[B19] Wennberg JE (1979). Factors governing utilization of hospital services. Hospital Practice.

[B20] Knickman JR, Foltz AM (1984). Regional differences in hospital utilization. How much can be traced to population differences?. Med Care.

[B21] Wennberg J, Freeman JL, Culp WJ (1987). Are hospital services rationed in New Haven and over-utilized in Boston?. Lancet.

[B22] Paul-Shaheen P, Clark JD, Williams D (1987). Small area analysis: a review and analysis of the North American literature. J Health Polit Policy Law.

[B23] Stano M (1993). Evaluating the policy role of the small area variations and physician practice style hypotheses. Health Policy.

[B24] McMahon LFJ, Wolfe RA, Griffith JR, Cuthbertson D (1993). Socioeconomic influence on small area hospital utilization. Med Care.

[B25] Veugelers PJ, Hornibrook S (2002). Small area comparisons of health: applications for policy makers and challenges for researchers. Chronic Dis Can.

[B26] Pekoz EA, Shwartz M, Iezzoni LI, Ash AS, Posner MA, Restuccia JD (2003). Comparing the importance of disease rate versus practice style variations in explaining differences in small area hospitalization rates for two respiratory conditions. Stat Med.

[B27] Detsky AS (1995). Regional variation in medical care. N Engl J Med.

[B28] Makuc DM, Haglund B, Ingram DD, Kleinman JC, Feldman JJ (1991). The use of health service areas for measuring provider availability. J Rural Health.

[B29] Garnick DW, Luft HS, Robinson JC, Tetreault J (1987). Appropriate measures of hospital market areas. Health Serv Res.

[B30] Goodman DC, Wennberg JE (1999). Maps and health: the challenges of interpretation. Journal of Public Health Management & Practice.

[B31] Wennberg JE, Gittelsohn A (1980). A small area approach to the analysis of health system performance. Health Planning Methods and Technology Performance.

[B32] Goodman DC, Mick SS, Bott D, Stukel T, Chang CH, Marth N, Poage J, Carretta HJ (2003). Primary care service areas: a new tool for the evaluation of primary care services. Health Serv Res.

[B33] Ricketts TC, Randolph R, Howard HA, Pathman D, Carey T (2001). Hospitalization rates as indicators of access to primary care. Health Place.

[B34] Zwarenstein M, Krige D, Wolff B (1991). The use of a geographical information system for hospital catchment area research in Natal/KwaZulu. S Afr Med J.

[B35] Zwakhals SL, Giesbers H, Mac Gillavry E, van Boven PF, van der Veen AA (2004). The Dutch National Atlas of Public Health. Bundesgesundheitsblatt Gesundheitsforschung Gesundheitsschutz.

[B36] Rytkonen M, Rusanen J, Nayha S (2001). Small-area variation in mortality in the city of Oulu, Finland, during the period 1978--1995. Health Place.

[B37] Benach J, Yasui Y, Borrell C, Rosa E, Pasarin MI, Benach N, Espanol E, Martinez JM, Daponte A (2003). Examining geographic patterns of mortality: the atlas of mortality in small areas in Spain (1987-1995). Eur J Public Health.

[B38] Garcia-Gil C, Cruz-Rojo C, Alvarez-Giron M, Solano-Pares A (2004). Health inequalities in Seville, Spain: use of indicators of social deprivation and mortality in small areas. Public Health.

[B39] Lichtner S, Pflanz M (1971). Appendectomy in the Federal Republic of Germany: epidemiology and medical care patterns.. Medical Care.

[B40] Mackenbach JP, Kunst AE, Looman CW, Habbema JD, van der Maas PJ (1988). Regional differences in mortality from conditions amenable to medical intervention in The Netherlands: a comparison of four time periods. Journal of Epidemiology & Community Health.

[B41] Mackenbach JP, Kunst AE, Looman CW (1991). Cultural and economic determinants of geographical mortality patterns in The Netherlands. Journal of Epidemiology & Community Health.

[B42] Kunst AE, Looman CW, Mackenbach JP (1993). Determinants of regional differences in lung cancer mortality in The Netherlands. Social Science & Medicine.

[B43] Ubido J, Ashton J (1993). Small area analysis: abortion statistics. Journal of Public Health Medicine.

[B44] Katalinic A, Bartel C, Uhlenkamp T, Raspe H (1999). [Developing a small-area cancer atlas: process, validity and possible applications]. Gesundheitswesen.

[B45] Gatrell A, Lancaster G, Chapple A, Horsley S, Smith M (2002). Variations in use of tertiary cardiac services in part of North-West England. Health Place.

[B46] Schober E, Rami B, Waldhoer T (2003). Small area variation in childhood diabetes mellitus in Austria: links to population density, 1989 to 1999. J Clin Epidemiol.

